# Skin picking: A problem among healthcare workers?

**DOI:** 10.1002/hsr2.1348

**Published:** 2023-06-14

**Authors:** Cara Symanzik, Richard Brans, Christoph Skudlik, Swen M. John, Katja Dicke

**Affiliations:** ^1^ Department of Dermatology, Environmental Medicine and Health Theory Osnabrück University Osnabrück Germany; ^2^ Institute for Interdisciplinary Dermatological Prevention and Rehabilitation (iDerm) at Osnabrück University Osnabrück Germany

**Keywords:** dermatillomania, excoriation disorder, healthcare workers, nurses, pathological skin picking, skin picking

Skin picking (SP) with manipulation of the skin using nails or other tools leads to various degrees of skin damage. Clinical signs of SP on the hands include erythema, erosions and bleeding wounds, crusts, hyperkeratosis, and often loss of the cuticles^1^, ^2^ (Figure 1).

**Figure 1:**
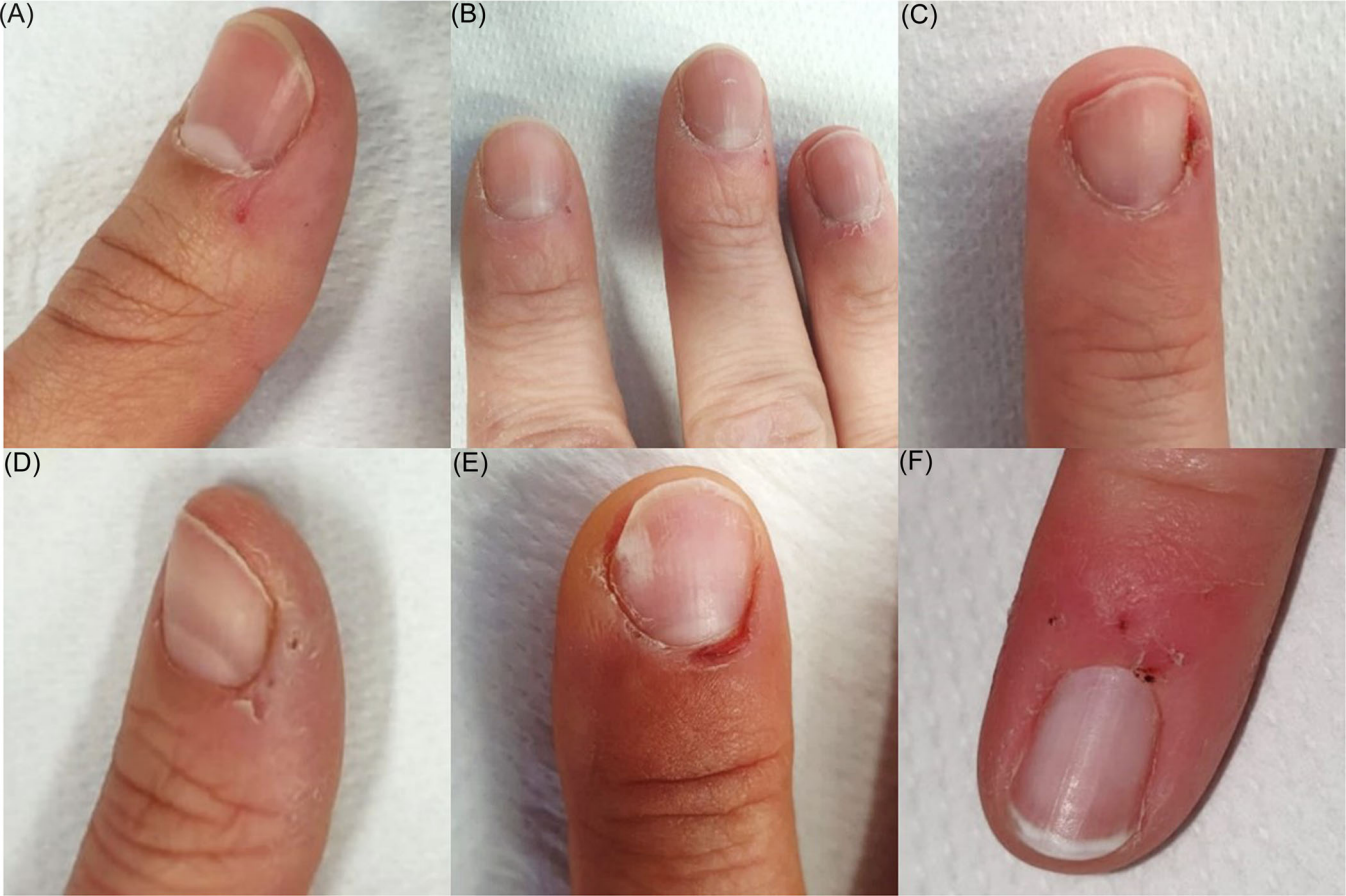
Skin changes in study participants attributable to skin picking: (A) Hyperkeratoses at the nail fold, loss of the cuticle, light periungual erythema with erosion at the digitus (dig.) I of the right hand; (B) light periungual erythema with marginal scales and individual erosions of the dig. II–IV of the right hand and hyperkeratoses on the nail fold and nail wall, loss of the cuticle; (C) loss of cuticle, light periungual erythema, hyperkeratosis of nail wall and nail fold, erosion of nail wall of dig. III right hand; (D) periungual erythema with fine to medium lamellar scaling and isolated small crusts on the right dig. I; (E) hyperkeratoses at the nail fold, loss of the cuticle and light periungual erythema with evidence of erosion at the nail fold of the dig. III right hand; (F) loss of the cuticle and periungual erythema of the dig. V of the left hand.

The manipulation ranges from relatively normal behavior to self‐injurious pathological SP related to compulsive‐impulsive behavior or other psychiatric comorbidities causing functional interferences or distress.^3^, ^4^ Focusing on the general population, the lifetime prevalence for pathological SP is estimated at 1.4%–5.2%^3^ and might be even higher in patients with skin diseases.^5^ The current paper aims at orientationally assessing the occurrence of clinical signs of SP on the hands of healthcare workers (HCWs).

In two hospitals in Lower Saxony, the skin condition on the hands of HCWs, including signs of SP, was dermatologically assessed in December 2020 and January 2021 within the framework of other examinations, which are described elsewhere.^6^ It was recorded by the investigating dermatologists when clinical signs of SP on the hands were visible (yes/no). Ethic approval was obtained by the sub‐commission on the evaluation of medical research involving human subjects at the Medical Chamber of Lower Saxony, Hannover, Germany (procedure number 30/34/2020). Informed consent was obtained by all participants. The trial was prospectively registered at the German Clinical Trials Register (DRKS) (number DRKS00022957). Data for the presented work were analyzed in terms of descriptive statistics.

A total of 302 HCWs participated (84.1% female, mean age 39.0 ± 12.7 years). The majority were nurses (55.3%). Skin lesions attributable to SP were present in 19.5% of the 302 HCWs. The skin lesions recorded were primarily localized periungually; in most cases, there was a loss of the cuticle due to active manipulation (Figure 1).

The 19.5% proportion of HCWs found in the present work who show signs of SP can generally be described as high. Since there have only been a few studies on the subject of SP to date, there is a lack of comparable data. In daily practice, SP might often be overlooked, which could be due to the often small lesions, a lack of knowledge about the clinical picture, and a lack of inclusion in standard clinical diagnostics.

This was an explorative study. We were unable to differentiate between relatively normal and pathological SP. In future studies, comprehensive psychological assessments could accompany clinical examinations to enable this differentiation. Furthermore, it would be of interest to assess the severity of lesions and potential associations with stress or other factors (e.g., knowledge level of HCWs about skin care and protection), which was not investigated in this study.

It can be assumed that the highest share of clinical signs of SP in this cohort is not attributable to underlying psychiatric disorders but rather to removing bothersome flaps of skin as a common human behavior; although, the transitions might be fluid. HCWs frequently suffer from dry skin and irritant contact dermatitis on their hands due to wet work, which may promote SP. Dry, scaly skin, especially periungual, could trigger the need to pluck it off. Regular skin care and protection measures could therefore be of great importance to reduce SP in HCWs. Stress is known to be one of the main trigger factors of pathological SP^7^ and may have contributed to the high proportion of SP in this cohort. In pathological SP, behavioral therapy is advised to reduce or avoid undesirable behavior.^3^ In view of the fact that skin lesions can also represent an entry point for pathogens, greater attention should be paid to also minor skin lesions and their avoidance, especially in HCWs. To minimize recurrent skin lesions in HCWs, it is considered sensible to counteract factors that may promote and/or intensify the occurrence of SP with preventive measures. Further research is needed on the topic of SP among HCWs to adequately design and implement diagnostic as well as preventive measures in the future.

## AUTHOR CONTRIBUTIONS

**Cara Symanzik**: conceptualization; data curation; formal analysis; investigation; methodology; project administration; visualization; writing—original draft; writing—review & editing. **Richard Brans**: conceptualization; methodology; writing—review & editing. **Christoph Skudlik**: funding acquisition; writing—review & editing. **Swen M John**: conceptualization; funding acquisition; methodology; project administration; resources; supervision; writing—review & editing. **Katja Dicke**: conceptualization; methodology; writing—original draft; writing—review & editing.

## CONFLICT OF INTEREST STATEMENT

The authors declare no conflicts of interest.

## FUNDING INFORMATION

This study was supported by a restricted monetary and material donation (study products) from Beiersdorf AG, Hamburg, Germany. Beiersdorf AG had no role in study design, data collection and analysis, decision to publish, or preparation of the manuscript.

## ETHICS STATEMENT

Ethic approval was obtained by the sub‐commission on the evaluation of medical research involving human subjects at the Medical Chamber of Lower Saxony, Hannover, Germany (procedure number 30/34/2020). Informed consent was obtained by all participants. The trial was prospectively registered at the German Clinical Trials Register (DRKS) (number DRKS00022957).

## TRANSPARENCY STATEMENT

The lead author Dr. Cara Symanzik affirms that this manuscript is an honest, accurate, and transparent account of the study being reported; that no important aspects of the study have been omitted; and that any discrepancies from the study as planned (and, if relevant, registered) have been explained.

## Data Availability

The data that support the findings of this study are available from the corresponding author upon reasonable request.
